# Ultrasound-Activated Piezoelectric Nanoparticles Inhibit Proliferation of Breast Cancer Cells

**DOI:** 10.1038/s41598-018-24697-1

**Published:** 2018-04-19

**Authors:** Attilio Marino, Matteo Battaglini, Daniele De Pasquale, Andrea Degl’Innocenti, Gianni Ciofani

**Affiliations:** 1 Istituto Italiano di Tecnologia, Smart Bio-Interfaces, Viale Rinaldo Piaggio 34, 56025 Pontedera, Italy; 2 Scuola Superiore Sant’Anna, The Biorobotics Institute, Viale Rinaldo Piaggio 34, 56025 Pontedera, Italy; 3 Politecnico di Torino, Department of Mechanical and Aerospace Engineering, Corso Duca degli Abruzzi 24, 10129 Torino, Italy

## Abstract

A nanotechnology-based approach for the inhibition of breast cancer cell proliferation is proposed. The innovative solution consists in a platform based on biocompatible piezoelectric nanoparticles able to target and remotely stimulate HER2-positive breast cancer cells. The anti-proliferative effects of the ultrasound-driven piezoelectric nanoparticle-assisted stimulation significantly reduced the proliferation by inducing the cell cycle arrest. Similarly to a low-intensity alternating electric field, chronic piezoelectric stimulation resulted able to inhibit cancer cell proliferation by upregulating the expression of the gene encoding Kir3.2 inward rectifier potassium channels, by interfering on Ca^2+^ homeostasis, and by affecting the organization of mitotic spindles during mitosis. The proposed platform, even if specific for HER2-positive cells, shows huge potential and versatility for the treatment of different type of cancers.

## Introduction

Breast cancer is the most widespread cancer and the second most common cause of cancer death in women^1^. In addition to surgery, several pre/post-operative treatments, such as drug/hormone therapy, radiotherapy and chemotherapy, are adopted depending on the specific stage and on the type of the tumor. The heterogeneity of breast cancers regards tumor grade, lymph node status, histological properties, and molecular profile. According to expression of markers like human epidermal growth factor type 2 receptor (HER2), estrogen receptor, and progesterone receptor, breast cancers are classified into five subtypes: HER2, luminal A, luminal B, basal, and normal^2,3^. A significant percentage of breast cancers (between 20–30%) expresses high levels of HER2^4^. These tumors are called HER2-positive breast cancers. Epidermal growth factor (EGF) is known to specifically bind the HER2 receptor of cancer cells, so stimulating them to proliferate and to form metastasis. HER2-positive breast cancers are characterized by higher growth rates and by a higher probability to generate metastases and to invade other tissues with respect to HER2-negative breast cancers. Different drugs, such as trastuzumab, lapatinib, and pertuzumab, have been developed in order to interfere with the EGF-HER2 pathway. In particular, trastuzumab is a monoclonal antibody that specifically binds HER2 receptor, and it is the most common drug used in the targeted therapy against HER2-positive breast cancers^5^. Studies with trastuzumab showed as this drug is able to reduce the risk of cancer reoccurrence in early-stage HER2-positive breast cancers, and to improve the overall survival in metastatic late-stage ones^6,7^. However, there is an urgent need to develop new strategies to overcome the acquired resistance to chemotherapy, which is increasingly occurring among patients^8^.

Low-intensity electric stimulation represents an alternative treatment able to inhibit the proliferation of different tumor cell lines^9^. Specifically, low intensity electric stimulation inhibits cell division by affecting K^+^ channels (by inducing an overexpression of the inward rectifier K^+^ channel Kir3.2)^10^ and by interfering with cytoskeletal structures during the cell division (in particular with a disorganization of the mitotic spindle)^11^. Furthermore, low-intensity and low-frequency alternating current (AC) stimulation resulted able to significantly enhance the effects of chemotherapy for the treatment of glioblastoma in clinical trials^12^. Specifically, AC not only is able to affect cancer cell proliferation without the use of any drugs, yet also reduce multidrug resistance by impairing the plasma membrane translocation of MDR1, a P-glycoprotein (P-gp) the overexpression of which is associated to chemotherapy resistance^13^. Nevertheless, AC stimulation can also affect the proliferation of non-malignant cells (*i.e*., human astrocytes)^10^ and, for this reason, the specific and targeted delivery of electric stimuli just to cancer cells is of extreme importance.

Piezoelectric nanomaterials have shown to be a very promising tool for a wireless, non-invasive, and targeted electric stimulation of cells and tissues^13^. When these nanomaterials undergo mechanical deformations, they are able to generate electric potentials on their surface thanks to the direct piezoelectric effect. The remote activation of piezo-nanotransducers can be obtained by exploiting different sources of mechanical stimulation, such as, for example, ultrasounds (US)^14^. US are able to efficiently penetrate biological tissues in a non-harmful way, and are widely exploited in medicine for diagnostic imaging. In the context of ultrasonography, different nanoparticles have been also adopted as long-lasting US contrast agents for diagnostic purposes^15,16^.

Recently, findings of our group reported the successful remote stimulation of different cell types by synergistically exploiting biocompatible piezoelectric barium titanate nanoparticles (BTNPs) in combination with US^17–20^. In this work, we describe an innovative piezoelectric nanoplatform consisting in BTNPs functionalized with anti-HER2 antibody (Ab-BTNPs), able to specifically target HER2 positive cancer cells. Anti-proliferative effects of remote piezoelectric stimulation (US + Ab-BTNPs) on SK-BR-3 breast cancer cells were verified in terms of cell cycle progression analysis, expression of proliferation markers, and metabolic activity of cell cultures. Finally, analysis of intracellular Ca^2+^ levels, evaluation of mitotic spindle development, and assessment of K^+^ channel expression at gene level corroborated our findings.

## Methods

### Characterization of piezoelectric barium titanate nanoparticles

Piezoelectric barium titanate nanoparticles (BTNPs) with tetragonal crystalline configuration (Nanostructured & Amorphous Materials, Inc.) were coated with 1,2-distearoyl-*sn*-glycero-3-phosphoethanolamine-*N*-[methoxy(polyethylene glycol)-5000] (DSPE-PEG, Nanocs) in order to obtain a stable nanoparticle dispersion in aqueous solutions. 10 mg of plain BTNPs were dispersed in 10 ml of aqueous solution containing 1 mg/ml DSPE-PEG by using a tip sonicator (8 W for 150 s; Mini 20 Bandelin Sonopuls). Nanoparticles were then centrifuged to clean any residual DSPE-PEG (900 rcf for 20 min, Hettich®Universal 320/320 R centrifuge), and the pellet resuspended in 10 ml of ddH_2_O to obtain a 1 mg/ml stable BTNP dispersion.

Obtained dispersion was subsequently characterized through dynamic light scattering (DLS, Nano Z-Sizer 90, Malvern Instrument), scanning electron microscopy (SEM, Helios NanoLab 600i FIB/SEM, FEI), transmission electron microscopy (TEM, JEM-1011 microscope, JEOL, equipped with a 11 Mp Gatan Orius CCD camera and a tungsten thermionic gun operating at a 100 kV accelerating voltage), and thermogravimetric analysis (TGA Q500-TA Instrument).

DLS of the diluted BTNP dispersion (100 μg/ml) allowed measuring both *Z*-potential (average ± standard deviation of 24 runs) and hydrodynamic radius (average ± standard deviation of 12 runs).

For SEM imaging, a 50 μl drop of BTNP dispersion was deposited on a silica wafer and let dry for 4 h. Subsequently, samples were gold-sputtered at 60 nA for 25 s before SEM observation.

Concerning TEM and electron diffraction analyses, a 10 μl drop of the BTNP dispersion was placed on carbon-coated 200 mesh copper grids and allowed to air-dry before observation.

Finally, for the quantification of the DSPE-PEG coating, thermogravimetric analysis (TGA) of 15 mg of BTNP powder, DSPE-PEG powder, or lyophilized DSPE-PEG-BTNPs has been carried out under nitrogen (flow rate of 50 ml min^−1^) at a heating rate of 5 °C min^−1^ from 30 to 500 °C.

### Functionalization of BTNPs with anti-HER2 antibody (Ab-BTNPs)

The functionalization of BTNPs with antibody against HER2 was obtained by exploiting the biotin-streptovidin conjugation. Specifically, 10 mg of plain BTNPs were mixed with 10 ml of aqueous solution containing 0.8 mg/ml DSPE-PEG and 0.2 mg/ml DSPE-PEG-biotin, and thereafter dispersed by using a tip sonicator as previously described. Nanoparticles were then rinsed through centrifugation, and a 10 mg/ml stable dispersion of biotin-BTNPs was obtained. Subsequently, 25 µl of 1 mg/ml streptovidin-Ab (AC17-0032-22, Abcore) were mixed with the biotin-BTNP suspension for 90 min at 4 °C under shaking. After functionalization, the unbound Ab was dialyzed overnight. The estimation of the functionalization efficiency was directly performed on Ab-BTNPs through the bicinchoninic acid (BCA) assay (Thermo Fisher) by following the enhanced test tube protocol as indicated by the manufacturer.

### Cell culture and BTNP treatment

SK-BR-3 cells (ATCC® HTB-30™) were adopted as *in vitro* model of HER2-positive human breast cancer. SK-BR-3 cell cultures develop grape-like and stellate structures and show a more invasive phenotype *in vitro* with respect to other breast cancer cell lines, such as T47D, MCF-7, or BT474^21^. Molecular profile of SK-BR-3 cells has been intensively characterized by immunohistochemistry analysis, which highlighted not only a remarkable overexpression of HER2, but also a higher expression of another important marker of breast cancer, epidermal growth factor receptor (EGFR), with respect to other HER2-positive cell lines (*e.g*., MDA-MD-435, MDA-MD-435 and BT-474)^22^. Cells were cultured in T-75 flasks by using McCoy’s 5A (modified) medium (Thermo Fisher Scientific), supplemented with 10% fetal bovine serum (FBS, Gibco), 100 IU/ml penicillin (Gibco), 100 μg/ml streptomycin (Gibco). For the experiments, cells were seeded in 35 mm diameter μ-dishes (Ibidi) at a density of 7500 cell/cm^2^.

In order to investigate the anti-proliferative effects of anti-HER2, SK-BR-3 cells were treated with different concentrations (0–100 µg/ml) of anti-HER2 Ab for 3 and 5 days. Thereafter, cell metabolism was assessed through WST-1 assay ((2-(4-iodophenyl)-3-(4-nitophenyl)-5-(2,4disulfophenyl)-2H-tetrazoilium monosodium salt, provided in a premix electrocoupling solution, BioVision) as described elsewhere^23^. The absorbance was read at 450 nm with a microplate reader (Victor3, PerkinElmer). The metabolism of the different experimental conditions was then expressed by normalizing the absorbance values for those of control cultures.

WST-1 assay was also performed to evaluate the toxicity of BTNPs and Ab-BTNPs at different nanomaterial concentrations (0–250 µg/ml; 300 µl of incubation volume) after 5 days of treatment. For all of the following experiments, a concentration of 100 µg/ml of BTNPs or Ab-BTNPs was used as it resulted to be non-toxic and to be adequate to elicit cellular response after US stimulation^18,24^. Considering that (*i*) *per* each well the total amount of incubated nanoparticles was 33 µg, (*ii*) after 24 h the total amount of nanomaterial resulted deposited on the surface of the cell culture, and (*iii*) ~25000 cells were seeded on a 3.5 cm^2^ area with a confluence of ~10%, an average of ~1000 BTNPs are estimated to be associated to each cell.

### Nanoparticle-cell interaction analysis

To evaluate the nanoparticle targeting on breast cancer cells, SK-BR-3 cultures were treated for 1 h with BTNPs or Ab-BTNPs, subsequently washed twice with PBS and stained with the CellMask Green Plasma Membrane Stain (1:1000 dilution, Invitrogen) in serum-free Dulbecco’s Modified Eagle’s Medium (DMEM, Thermo Fisher) at 37 °C. The staining solution was removed, samples were rinsed with PBS and finally incubated with HEPES-supplemented (25 mM) phenol red-free DMEM (Thermo Fisher) for confocal laser scanning confocal microscopy (CLSM, C2s system, Nikon). BTNPs were detected by using a laser excitation of 642 nm and by collecting the emission from 670 to 750 nm, as showed elsewhere^18,20,24^. 3D rendering of *z*-stacks was performed by using NIS-Elements software (Nikon).

Furthermore, SEM imaging of nanoparticle-incubated cells was carried out in combination with energy-dispersive X-ray (EDX) spectroscopy. Paraformaldehyde-fixed samples were treated with 2.5% glutaraldehyde solution for 30 min at 4 °C. Subsequently, gradual sample dehydration was obtained through progressive ethanol gradients (0%, 25%, 50%, 75%, and 100% in ddH_2_O). Samples were gold-sputtered as previously described and then observed with SEM (Helios NanoLab 600i FIB/SEM, FEI).

Nanoparticle targeting was also quantitatively investigated on SK-BR-3 cells treated for 1 h with BTNPs or Ab-BTNPs through inductively coupled plasma mass spectroscopy (ICP). For ICP analysis, cells were cultured and treated with nanoparticles in T75 flasks. After 1 h of nanoparticle incubation, cells were washed twice with PBS and detached with trypsin (5 min at 37 °C; Sigma Aldrich). The cell pellets were then analyzed by elemental analysis (ICP-OES spectrometer, iCAP 6500, Thermo). A concentrated HCl/HNO_3_ (Sigma Aldrich super-pure grade) solution (1 ml) was added to the known amount of cell pellets. After 24 h, 9 ml of MilliQ grade water was then added to the sample. The 233.5 nm barium emission line and the 334.9 nm titanium emission line were used.

For the investigation of nanoparticle internalization, SK-BR-3 cells were treated for 4 h and 24 h with BTNPs or Ab-BTNPs, and subsequently washed twice and stained with Lysotracker (75 nM, Invitrogen) and Hoechst 33342 (1 μg/ml, Invitrogen) in serum-free DMEM (Thermo Fisher) at 37 °C. CLSM imaging was then performed as described above.

### US stimulation and Ca^2+^ imaging

Chronic US stimulations were applied with a KTAC-4000 device (Sonidel) with a planar ultrasound transducer of 20 mm diameter by setting 0.2–1 W/cm^2^, 0.5 Hz burst rate, and 10% duty cycle. These US parameters were able to not induce any detectable temperature increments of the cell medium even after many hours of stimulation.

For Ca^2+^ imaging experiments, before US stimulation, cells were incubated for 30 min at 37 °C with Fluo-4 AM (1 μM, Invitrogen) and, subsequently, samples were rinsed with PBS and incubated with HEPES-supplemented (25 mM) phenol red-free DMEM (Thermo Fisher) for time-lapse fluorescence imaging (Eclipse Ti-E epifluorescence microscope, Nikon). Fluorescence intensities at different time points were calculated as mean value of pixels measured in intracellular region of interest (ROI), once subtracted the extracellular background. The average fluorescence intensity of the different ROIs was indicated as *F*_0_ at time *t* = 0 s, while for *t* > 0 s it was generically indicated as *F*. Graphs report the *F/F*_0_ traces related to different experimental conditions (US and US + Ab-BTNPs) during time-lapse experiment. Representative images at *t* = 0 s, *t* = 150 s, and *t* = 300 s of time-lapse Ca^2+^ imaging were processed by using ImageJ (http://rsbweb.nih.gov/ij/). In particular, images were double-smoothed and converted to *F/F*_0_ by using the divide function of the Math process after the background subtraction.

### Immunofluorescence and flow cytometry

Effects of chronic US stimulations (1 h per day for 4 days, in the presence or absence of Ab-BTNPs) on SK-BR-3 cell proliferation were monitored in terms of cell metabolism, analysis of cell cycle, expression of the proliferative marker Ki-67, and morphology of the mitotic spindles. Cell metabolism was estimated through WST-1 assay, as described above.

The expression of the Ki-67 and the morphology of mitotic spindles were investigated by immunocytochemistry followed by CLSM imaging. Immunofluorescence staining was performed as described elsewhere^23^. Briefly, after cell fixation (4% paraformaldehyde in PBS for 20 min at 4 °C), membrane permeabilization (Triton X-100 in 0.1% in PBS for 25 min at room temperature), and treatment with blocking solution (10% goat serum in PBS for 1 h), cultures were incubated with primary rabbit IgG anti-Ki-67 antibody (1:150 in 10% goat serum for 45 min at 37 °C; Millipore) or with primary mouse IgG anti-α-tubulin antibody (1:1500 in 10% goat serum for 45 min at 37 °C; Merck). Subsequently, cells were rinsed 5 times with 10% goat serum in PBS and then incubated with a staining solution of 10% goat serum in PBS containing TRITC-conjugated phalloidin (100 μM, Millipore), Hoechst 33342 (1 μg/ml, Invitrogen), and the secondary antibody. TRITC-conjugated secondary anti-rabbit antibody (1:200; Millipore) or FITC-conjugated secondary anti-mouse antibody (1:75; Millipore) were used, respectively, for Ki-67 and α-tubulin staining. Imaging was performed by using a CLSM system (C2s system, Nikon).

Concerning flow cytometry experiments, cell cycle was analyzed by using propidium iodide (PI, Sigma) staining of DNA and then measuring cell fluorescence emission (λ_ex_ 488 nm; λ_em_ 690 ± 50 nm). This protocol allows evaluating the cell cycle phase by measuring the DNA levels present in the cells: cells in gap0 and gap1 (G0/G1) phases present less DNA amount compared to those undergoing DNA synthesis (S), and even less with respect to those in mitosis (M) and in G2 phase^25^. Therefore, the different fluorescence signal of the cells can be associated to 3 different populations (G0/G1, S and G2/M). Before performing the analysis at flow cytometer, SK-BR-3 cells were detached from dishes with trypsin (5 min at 37 °C), fixed (4% paraformaldehyde in PBS for 15 min at 4 °C), permeabilized (Triton X-100 in 0.1% in PBS for 10 min at room temperature), and stained with PI (50 ng/ml in PBS). Finally, cells were centrifuged (10 min at 3000 rpm) and resuspended in 500 μl of PBS for flow cytometer analysis (CytoFLEX Flow Cytometer, Beckman Coulter, Inc.).

### Quantitative real-time reverse-transcription polymerase chain reaction (qRT-PCR)

Concerning qRT-PCR experiments, mature mRNA transcript of *KCNJ6* (encoding for Kir3.2) were investigated in control, US, and US + Ab-BTNPs experimental conditions as previously described^23^. Briefly, RNA was extracted through a phenol-chloroform procedure, purified (PCR purification kit, Qiagen), and subsequently quantified by using spectrophotometric analysis (NanoDrop, Thermo Scientific). Subsequently, a reverse transcription of 1 µg of mRNA was performed with a iScriptTM Reverse Transcription Supermix (Bio-Rad) by using the following temperature cycle: 25 °C for 5 min, 42 °C for 30 min, 42 °C for 30 min, and 85 °C for 5 min. cDNA amplification was obtained with a CFX Connect^TM^ Real-Time PCR Detection System (Bio-Rad) thermocycler and by using SsoAdvanced SYBR Green Supermix (Bio-Rad) with the following temperature protocol: 98 °C for 30 s (1 cycle), 98 °C for 3 s and 60 °C for 7 s (40 cycles), and a final temperature increase from 65 to 95 °C (1 cycle for melting curve generation). The reference gene was *RPL32*; for calculatin*g ΔΔC*_*t*_, cycle threshold (*C*_*t*_) of the “control” was used as reference. For *KCNJ6*, relevant sequence information was retrieved at Ensembl (human genome, GRCh38.p10)^26^; transcript features were then locally mapped on their genomic *locus*, *via* MacVector^27^. Candidate primer couples, aimed at detecting coding cDNA, were obtained with Primer^28^. To prevent amplification from genome, we imposed that at least one primer spans an exon-exon boundary. Final validations for quality and specificity were performed on NetPrimer (PREMIER Biosoft International; http://www.premierbiosoft.com/netprimer/index.html), UCSC *in silico* PCR^29^, and NCBI Blast^30^. Primer sequences are shown in Table S1.

### Image analysis and statistics

For statistic comparisons, normality of data distributions was tested with the Shapiro normality test and, subsequently, parametric ANOVA or non-parametric Kruskal-Wallis (KW) tests were performed depending on the normality of the data. After ANOVA and KW tests, Tukey’s HSD or Nemenyi-Damico-Wolfe-Dunn (NDWD) *post-hoc* tests were respectively carried out in order to compare all the single distributions. Finally, data were plotted as average ± standard deviation, in case of normal distributions, or as median ±95% confidence interval for non-normal distributions.

### Data availability Statement

The datasets generated and/or analyzed during the current study are available from the corresponding author on reasonable request.

## Results

### Functionalization of piezoelectric BTNPs for HER^+^ breast cancer targeting

**Figure 1:**
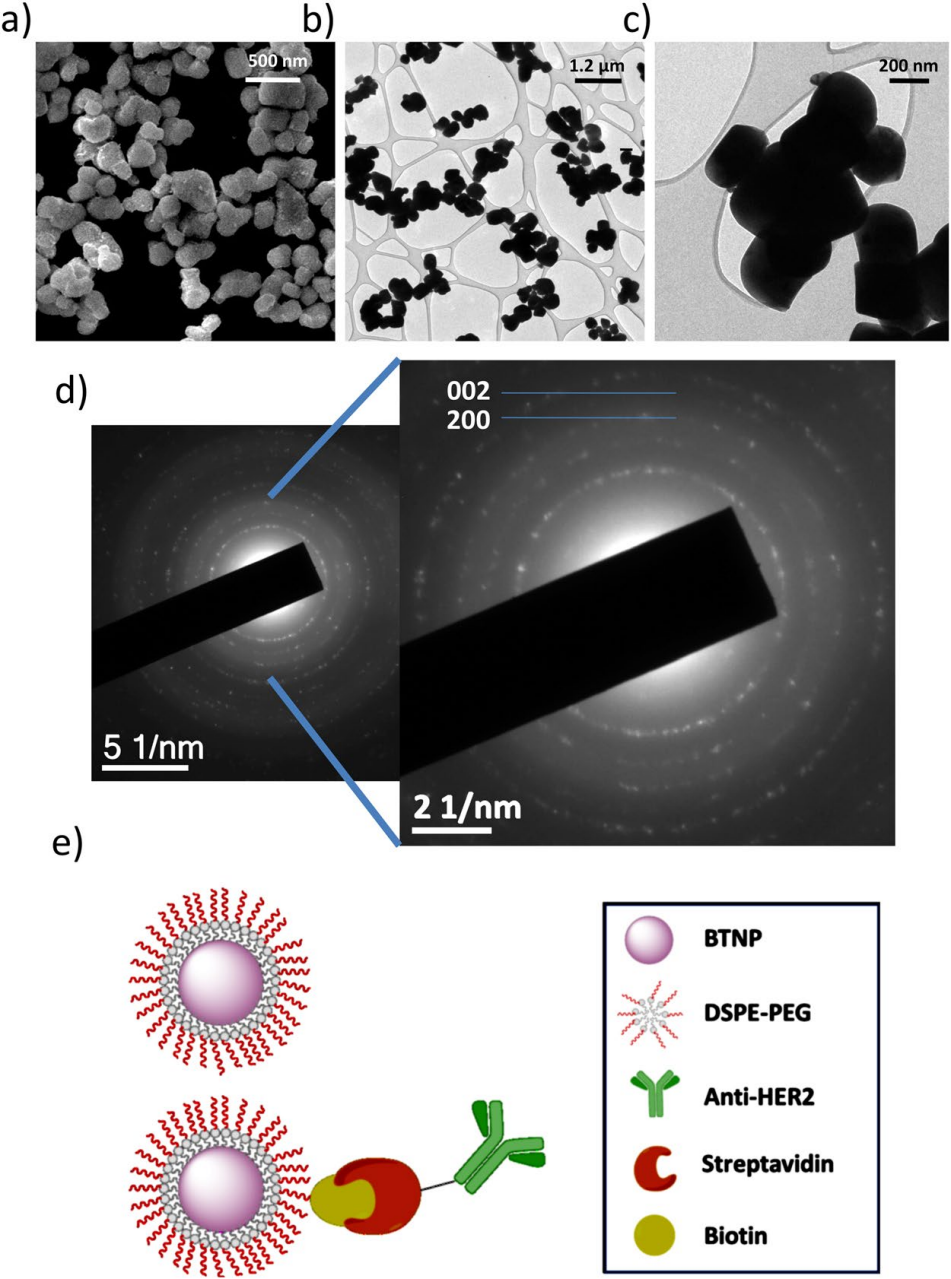
Characterization and functionalization of BTNPs. (**a**) SEM scan of BTNPs. TEM imaging at low (**b**) and high (**c**) magnification. (**d**) Diffraction pattern of BTNPs highlighting the typical double peaks at (200) and (002) of the tetragonal crystalline configuration. (**e**) Scheme of the functionalization of BTNPs with anti-HER2 antibody through DSPE-PEG-biotin coating.

**Figure 2:**
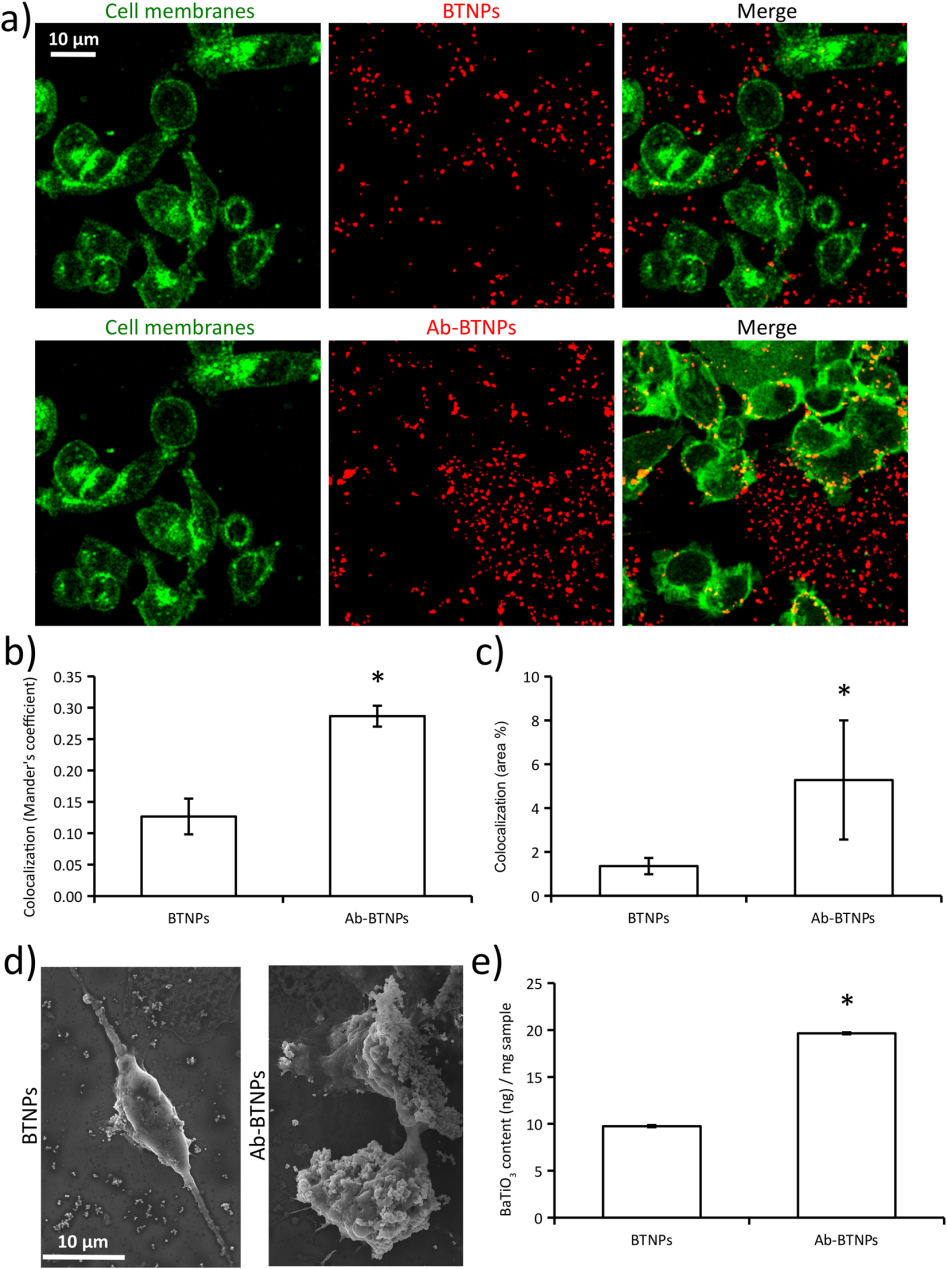
Analysis of BTNPs targeting to cancer cell membranes. (**a**) Confocal fluorescence microscopy of BTNPs/Ab-BTNPs associating with cancer cell membranes (SK-BR-3 plasma membranes in green, BTNPs in red, nuclei in blue). Histograms showing both (**b**) Mander’s coefficient and (**c**) area of BTNP signal overlapping with cell membrane signal. (**d**) SEM scan showing the surface of SK-BR-3 cells treated with BTNPs or Ab-BTNPS. (**e**) ICP analysis assessing BaTiO_3_ concentration in SK-BR-3 cultures treated for 1 h with BTNPs or Ab-BTNPs. (*p < 0.05).

The characterization of piezoelectric barium titanate nanoparticles (BTNPs) and the scheme of their functionalization through biotin-streptavidin conjugation are reported in Fig. 1. Representative SEM (Fig. 1a) and TEM (Fig. 1b) scans show typical round shape and homogeneous size of the nanoparticles. Their size (provided by the supplier to be 150 nm in radius) has been confirmed through high-magnification TEM image (Fig. 2c). The diffraction pattern of BTNPs, moreover, revealed the typical double peaks of tetragonal crystals at (200) and (002), thus confirming the piezoelectricity of the nanoparticles (Fig. 1d).

Figure 1e depicts the functionalization process of BTNPs. DSPE-PEG-coated BTNPs and Ab-streptavidin-biotin-DSPE-PEG-BTNPs will be indicated in the text, for easiness of reading, simply as BTNPs and Ab-BTNPs, respectively. Please, refer to “METHODS” for details about DSPE-PEG coating and Ab-conjugation.

The hydrodynamic radius and the *Z*-potential of BTNPs resulted of 198 ± 6 nm and −39.5 ± 0.4 mV, respectively (Figure S1a). The quantification of the DSPE-PEG coating through TGA indicated a 0.5% wt. of DSPE-PEG present in the BTNP sample (Figure S1b). Moreover, BCA measurements showed as 1.406 ± 0.004 µg/ml of Ab is conjugated to 1 mg/ml of Ab-BTNPs, demonstrating a good functionalization efficiency: since 25 µg of antibody were used for the conjugation and about 14 µg were found associated to BTNPs, more than 50% of the Ab used for the functionalization was linked to the BTNPs (about 220 molecules of Ab *per* BTNP).

### Ab-BTNPs efficiently target SK-BR-3 cells

Before assessing the targeting abilities of Ab-BTNPs, the anti-proliferative effects of anti-HER2 were tested on SK-BR-3 cells through WST-1 assay. Results showed that only relatively high concentrations (100 µg/ml) of anti-HER2 were able to significantly inhibit the proliferation of SK-BR-3 cells (Figure S2a; *p* < 0.05).

The WST-1 assays of SK-BR-3 cells treated with different concentrations (0–250 µg/ml) of BTNPs or Ab-BTNPs revealed as both the functionalized and non-functionalized nanoparticles are not eliciting any significant toxic effects after 5 days of incubation (Figure S2b; *p* > 0.05).

As already specified in the “METHODS”, despite all the tested nanoparticle concentrations resulted non-toxic, we decided to adopt a 100 µg/ml concentration for all the subsequent experiments since this was demonstrated to be enough to elicit significant responses in different cell types undergoing US stimulations^18,24^. Furthermore, 100 µg/ml dispersion of Ab-BTNPs are associated to 100 ng/ml of anti-HER2 Ab, which is remarkably safer compared to the potential anti-proliferative concentration (3 orders of magnitude lower). This condition enables to exploit the Ab just as nanomaterial targeting agent, and allows evaluating the anti-proliferative effects of the nanoparticle-assisted piezoelectric stimulations by excluding any synergic effect due to the presence of the antibody.

The ability of Ab-BTNPs to efficiently target cell membranes of SK-BR-3 cells after just 1 h of incubation was confirmed both through CLSM, SEM and ICP (Fig. 2). Qualitatively, a remarkable high amount of Ab-BTNPs was observed associated to the cell membranes of SK-BR-3 cells, while only few non-functionalized BTNPs were detected in correspondence of the cell membranes (CLSM imaging, Fig. 2a). The 3D rendering of CLSM imaging is showed in Supplementary Information (Figure S3). Quantitatively, both the Mander’s coefficient (Fig. 2b) and the % of BTNP signal overlapping with cell membrane signal (Fig. 2c) resulted significantly higher in case of Ab-BTNPs (Mander’s coefficient 0.29 ± 0.03; 5.28 ± 2.7% of overlapping signal) compared to the BTNPs without Ab (Mander’s coefficient 0.13 ± 0.02; 1.35 ± 0.37% of signal overlapping; *p* < 0.05).

SEM imaging revealed the presence of ~300 nanometer diameter particles on the surface of the SK-BR-3 cell membranes. The representative SEM scans of Fig. 2d also qualitatively show the higher amount of Ab-BTNPs associated to the plasma membranes of SK-BR-3 with respect to non-functionalized BTNPs. EDX analysis performed on the samples (Figure S4) showed co-localized presence of Ba (in green) Ti (in red) in correspondence to the particles on the cells; in details, the EDX spectrum highlighted the presence of the characteristic peaks of Ba (4.47 and 4.83 keV) and of Ti (4.51 keV).

According to these results, ICP quantitative analysis highlighted significantly higher concentrations of BaTiO_3_ in SK-BR-3 cultures treated with Ab-BTNPs (19.7 ± 0.1 ng/mg) with respect to those incubated with same concentration (100 µg/ml) of non-functionalized BTNPs (9.7 ± 0.1 ng/mg), further confirming the ability of the anti-HER2 Ab to target the piezoelectric nanomaterial towards HER2-positive cells (Fig. 2e).

**Figure 3:**
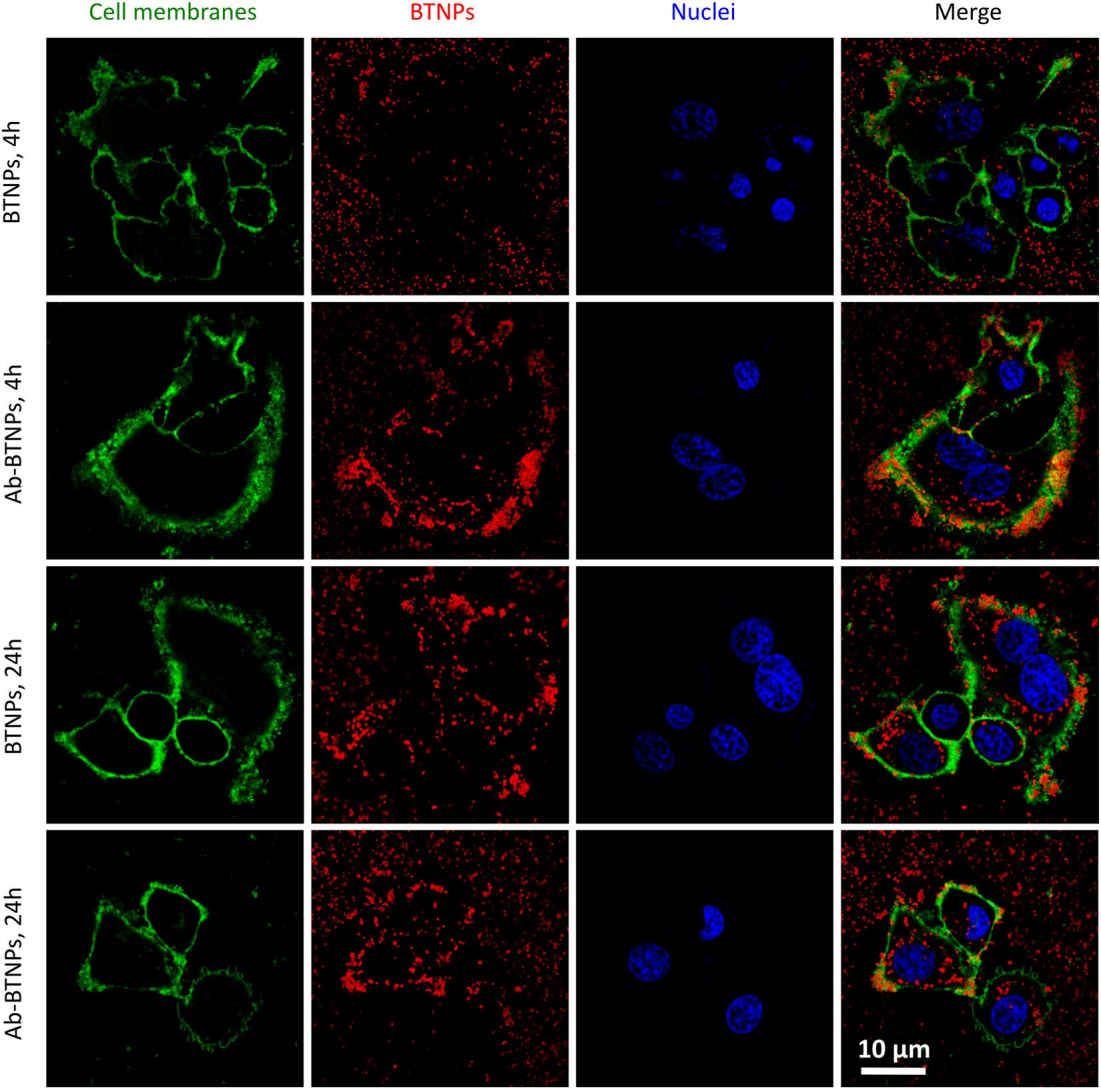
Confocal fluorescence imaging of BTNP uptake by SK-BR-3 cells after 4 h and 24 h of incubation (plasma membranes in green, BTNPs in red, nuclei in blue).

The nanoparticle internalization was investigated at 4 h and 24 h of incubation by staining cell membranes (Fig. 3) and lysosomes (Figure S5) of SK-BR-3 cells. Collectively, a higher amount of nanoparticles was observed associated to cell membranes in the case of Ab-functionalization, while the vast majority of the internalized nanoparticles (both for BTNPs and Ab-BTNPs) were observed into lysosomal compartments, thus suggesting that an endocytosis-mediated up-take of the nanomaterial is involved.

### Chronic piezoelectric stimulation promotes the exit of SK-BR-3 from cell cycle

The US-driven chronic piezoelectric stimulation was performed similarly as previously described^18,24^. A 200 ms US stimulus (frequency 1 MHz; power intensities from 0.2 to 1.0 W/cm^2^) was delivered every 2 s, 1 h *per* day, for 4 days. The duration of the single US stimulus was set at 200 ms, since during this period an effective cellular response occurrs^24^. The stimulus was delivered every 2 s to prevent any temperature increase of the cell medium. Similarly to electrical stimulation^10^, after 4 days of chronic piezoelectric stimulation a remarkable decrease of cancer cell proliferation was observed.

WST-1 analysis (Figure S6) showed a significant decrease of cell metabolism when stimulating SK-BR-3 cells for 4 days with US (1.0 W/cm^2^) + Ab-BTNPs (85 ± 3%; *p* < 0.05) compared to US (1.0 W/cm^2^) without nanoparticles (97 ± 10%) and to control cultures (not incubated with nanoparticles neither stimulated with ultrasounds, 100 ± 3%). No significant difference was moreover observed between control and US (1.0 W/cm^2^) cultures (*p* > 0.05), thus indicating that US chronic stimulation alone is not able to induce alteration of cell metabolism and/or cell proliferation. A non-significant trend of decreased proliferation activity was instead observed by stimulating Ab-BTNP-treated cells with US power intensities of 0.2 W/cm^2^ (97 ± 3%; *p* > 0.05) and 0.5 W/cm^2^ (92 ± 10%; *p* > 0.05). For this reason, all the following experiments have been performed at 1 W/cm^2^.

**Figure 4:**
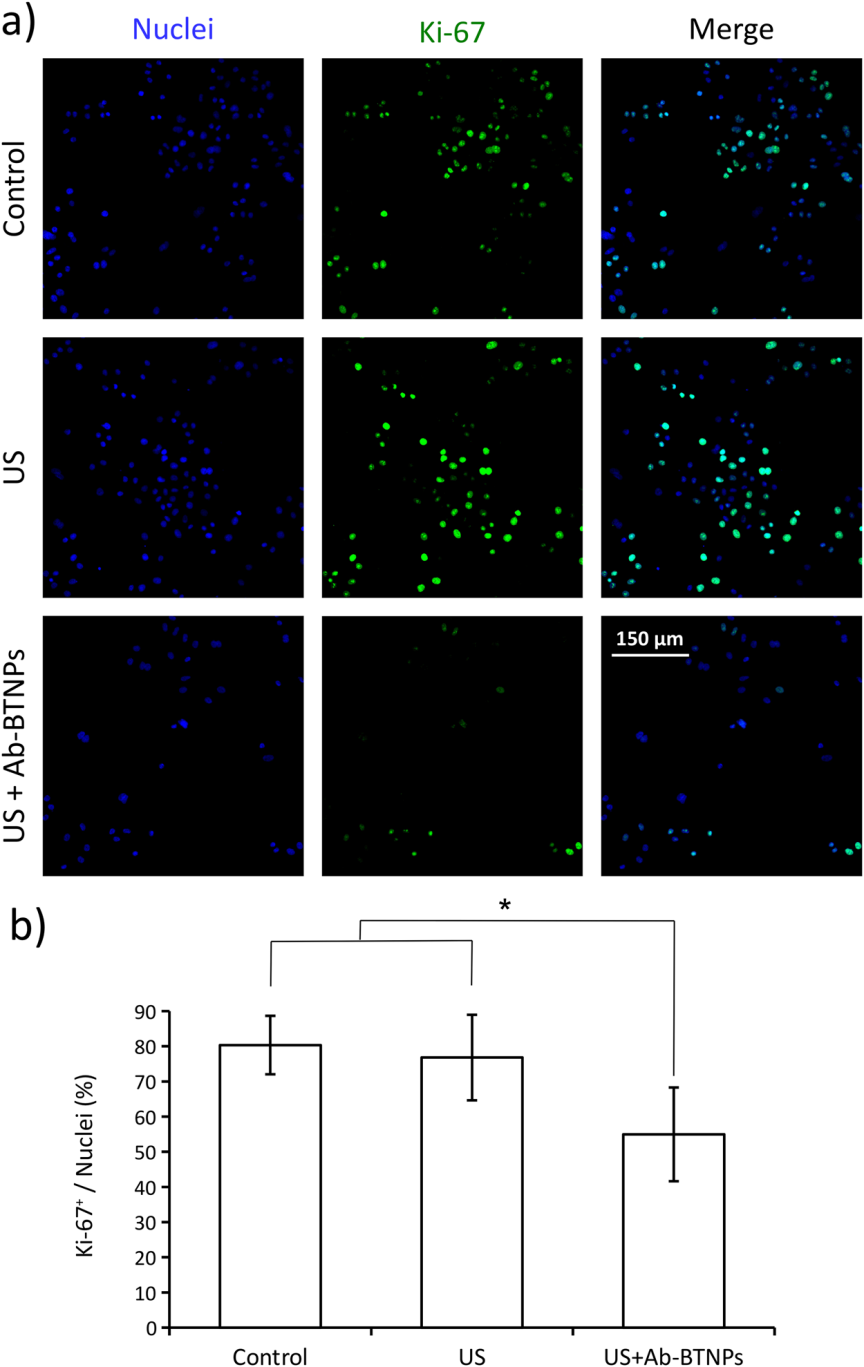
Effects of the chronic piezoelectric stimulation (US + Ab-BTNPs) on Ki-67 expression. (**a**) Confocal fluorescence images showing Ki-67 expression on control cultures, on ultrasound-stimulated cells without Ab-BTNPs (US), and on ultrasound-treated cultures after incubation with Ab-BTNPs (US + Ab-BTNPs); Ki-67 is shown in green, nuclei in blue. The percentages of Ki-67^+^ nuclei for each condition are shown in (**b**). *p < 0.05.

Consistently with WST-1 results, expression of Ki-67 proliferative marker was appreciably downregulated by the synergic US + Ab-BTNPs treatment (Fig. 4). In Fig. 4a nuclei (blue), Ki-67 (green), and the merged signals are reported for all the experimental conditions. These representative immunofluorescence images highlight a remarkable decrement of the Ki-67 expression in the US + Ab-BTNPs treated cultures with respect to the US and control groups. This observation was quantitatively confirmed: a lower percentage of cells expressing Ki-67 (Ki-67^+^ nuclei/total number of nuclei) was measured after the chronic piezoelectric stimulation (US + Ab-BTNPs, 56 ± 13%; *p* < 0.05) compared to both US (77 ± 12%) and to control (80 ± 8%) condition. The percentage of Ki-67^+^ nuclei was comparable in the US and control groups (*p* > 0.05). These results strongly suggest as the US-driven nanoparticle-assisted piezoelectric stimulation is able to promote the exit of the SK-BR-3 from the cell cycle.

**Figure 5:**
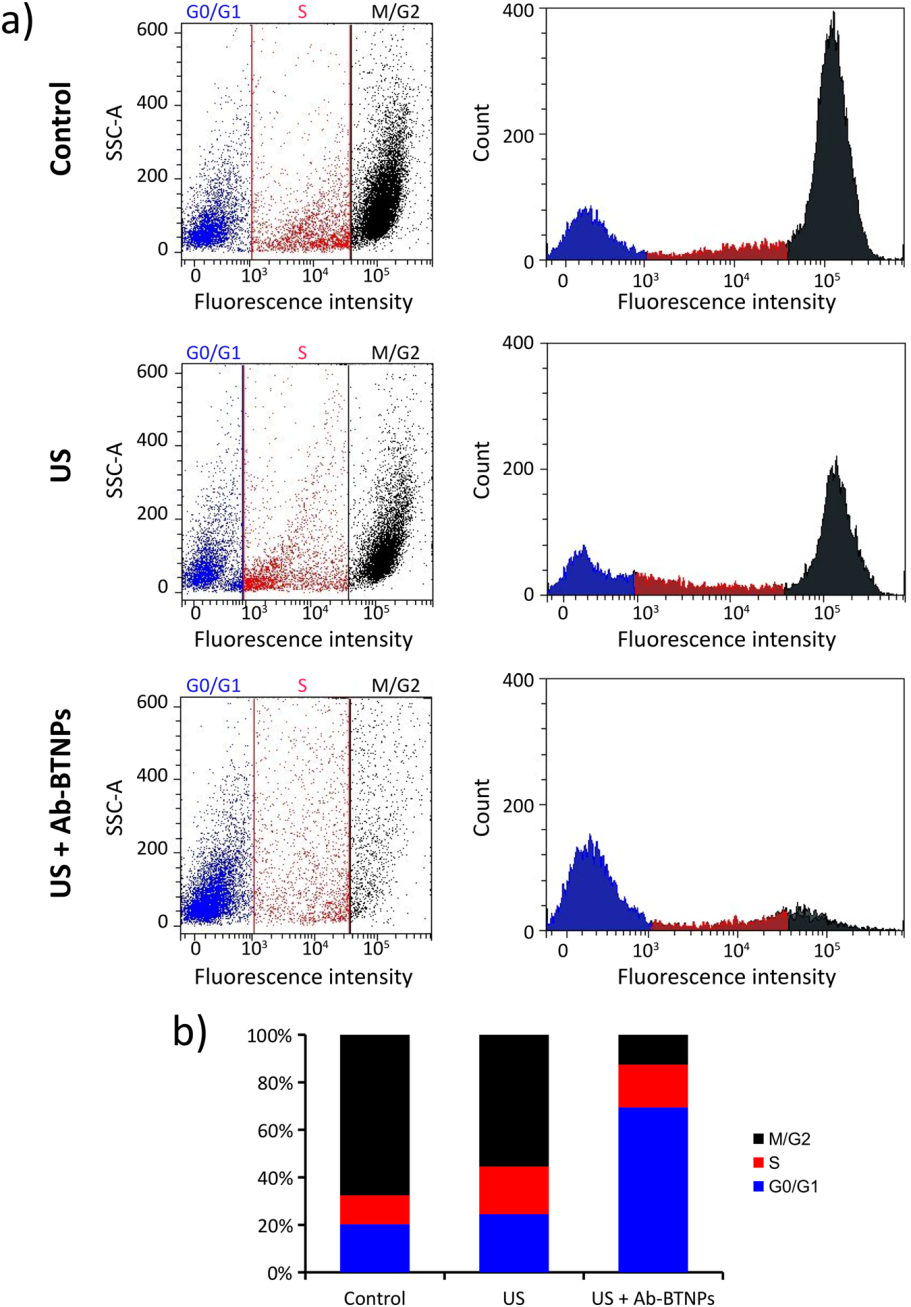
(**a**) Flow cytometry evaluation of cell cycle phases of SK-BR-3 cells treated with US, with chronic piezoelectric stimulation (US + Ab-BTNPs), or of control cultures by using propidium iodide staining (G0/G1 phases in blue; S phase in red; M/G2 phases in dark gray). The percentage of cells belonging to the different cell phases for each experimental condition is shown in (**b**).

To further confirm this hypothesis, the phase of cell cycle was analyzed by flow cytometry (Fig. 5; G0/G1 phases in blue, S phase in red, M/G2 phases in dark gray). Figure 5a reports, on the left, the side scattering (SSC-A) plot *vs*. the fluorescence intensity of the PI-stained SK-BR-3 cells in the three experimental groups. On the right, the graphs depict the counts as a function of the fluorescence intensity, showing the typical fluorescence peaks of G0/G1 (blue) and of M/G2 (dark gray) phases; the fluorescence signal between the two peaks (red) is associated to the cells in S phase. Concerning control and US experimental groups, it is possible to qualitatively appreciate a lower percentage of cells belonging to the G0/G1 population (blue), as most of cells are proliferating. Conversely, in the case of chronic piezoelectric stimulation (US + Ab-BTNPs), most of cells are in the G0/G1 (blue) gap, thus corroborating the hypothesis of exit from the cell cycle. The percentages of cells in the different cycle phases are reported in Fig. 5b. In the control group, 20.3% of cells are in G0/G1 phases, 12.2% in S phase, while most of cells (67.6%) are in M/G2 phases. In the US experimental group, 24.5% of cells are in G0/G1 phases, 20.1% of cells are in S phase, and most of cells (55.4%) are also in M/G2 phases. Finally, for cultures undergone US + Ab-BTNPs treatment, 69.5% of cells are in G0/G1 phases, 18.0% in S phase, and 12.5% in M/G2 phases.

**Figure 6:**
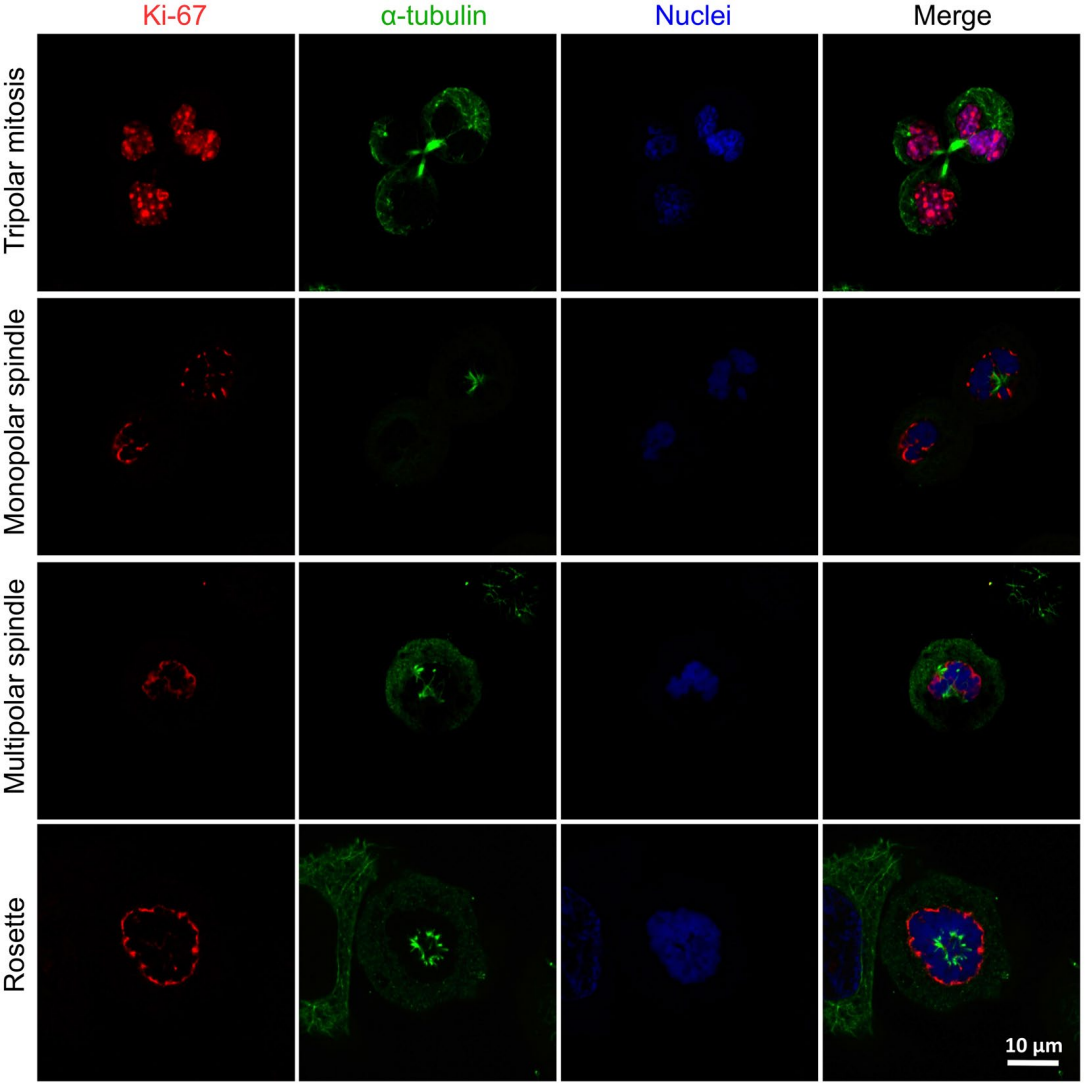
Confocal fluorescence images showing several abnormal mitotic conformations in SK-BR-3 cells after chronic piezoelectric stimulation: tripolar mitosis, monopolar spindle, multipolar spindle, and rosette (Ki-67 in red, α-tubulin in green, nuclei in blue).

AC stimulation is also known able to affect the mitosis process of cancer cells by interfering with mitotic spindle developing^11^. Interestingly, we observed a range of different abnormal mitotic conformations (*i.e*., monopolar spindle, tripolar mitosis, multipolar spindle, rosette) on piezoelectrically-stimulated cells (Fig. 6). These anomalous conditions, with exception of tripolar mitosis, were not detected on the other experimental conditions (US and control).

### Chronic piezoelectric stimulation upregulates gene expression of K^+^ channels and increases intracellular Ca^2+^ levels

**Figure 7:**
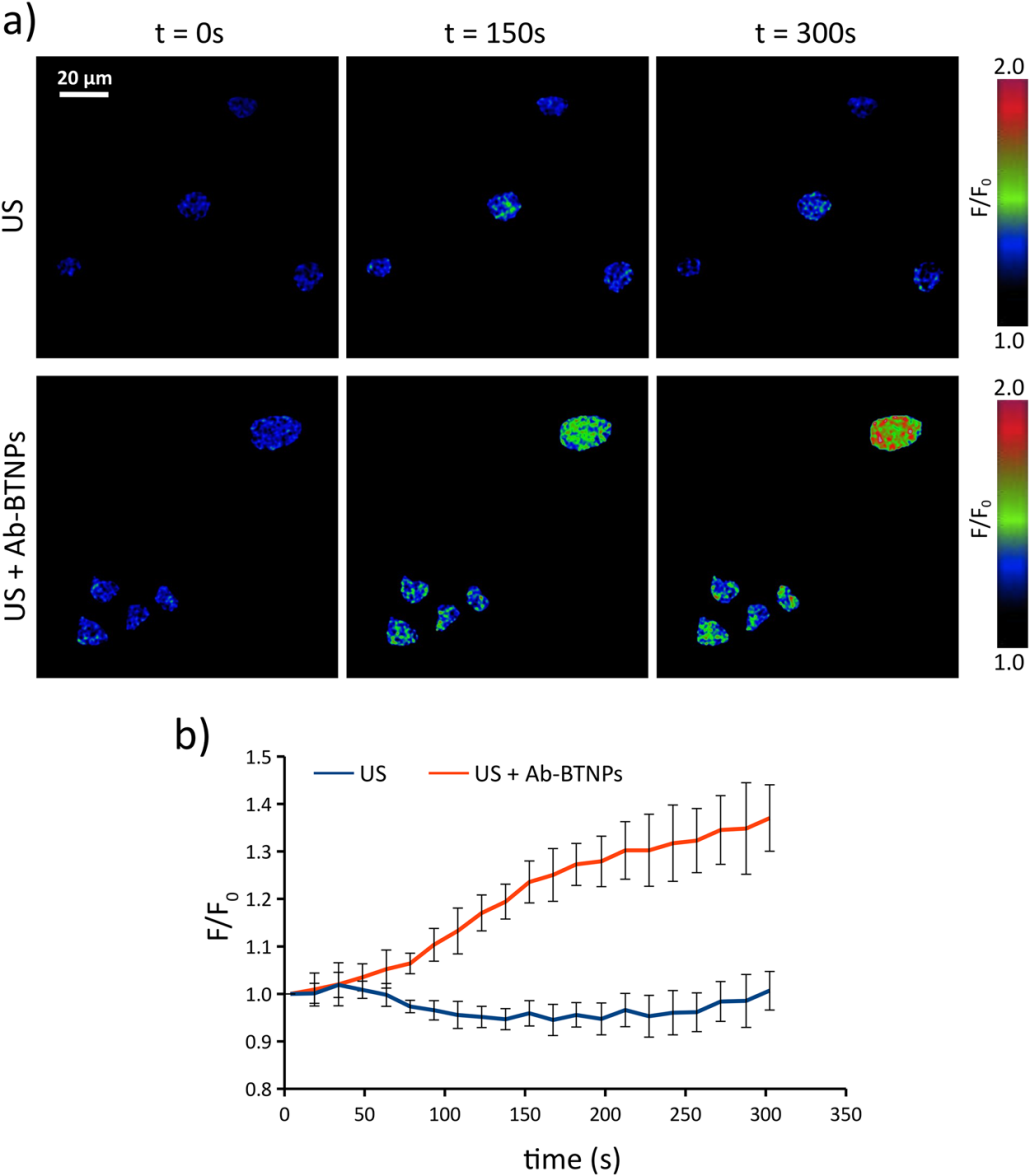
Analysis of intracellular Ca^2+^ levels during chronic ultrasounds (US) or piezoelectric (US + Ab-BTNPs) stimulation. (**a**) Representative *F*/*F*_0_ time-lapse Ca^2+^ imaging frames (*t* = 0 s; *t* = 150 s; *t* = 300 s). (**b**) *F/F*_0_ trends (**b**).

The mechanism mediating the electric stimulation-dependent inhibition of tumor proliferation involves an upregulation of the Kir3.2 inward rectifier K^+^ channels^9,10^. Unfortunately, given the reduced stimulation area, cell populations in our experimental conditions are too limited to allow the obtainment of enough biological material to carry out analysis at protein level, for example with the aid of Western blotting. To overcome this issue, expression of *KCNJ6* (encoding for Kir3.2) was quantitatively evaluated in terms of mature mRNA, which is a spliced and processed transcript ready for its translation into protein. Relative mRNA expression of *KCNJ6* gene in response to the US + Ab-BTNPs treatment indicates a significant (*p* < 0.05) upregulation (7.0 ± 2.0 folds) in response to the chronic piezoelectric stimulation, with respect to both the US stimulation (1.4 ± 0.2 folds) and the control cultures (1.0 ± 0.6). No significant difference on mRNA expression of *KCNJ6* was found between controls and US-stimulated cultures (*p* > 0.05). Additionally to Kir channels, Ca^2+^ homeostasis plays an important role on cancer cell proliferation, and its alteration contributes to the cell cycle arrest^31^. Since low-intensity electromagnetic fields are able to reduce cancer cell proliferation by inducing intracellular Ca^2+^ increments^32^, the effects of chronic piezoelectric stimulations on Ca^2+^ levels were studied through time-lapse Ca^2+^ imaging (Fig. 7). Figure 7a shows typical *F/F*_0_ time-lapse frames (*t* = 0 s; *t* = 150 s; *t* = 300 s) of cells stimulated with US both in absence and in presence of Ab-BTNPs. The US stimulation started at time *t* = 50 s. In Fig. 7b, the graphs report the traces of *F/F*_0_ related to the US (in blue) and to the US + Ab-BTNPs (in red) treatments, and highlight a significant increase of intracellular Ca^2+^ levels when US are applied in the presence of the piezoelectric nanoparticles. No Ca^2+^ increases were instead detected in response to the US stimulation without the presence of Ab-BTNPs.

## Discussion

In this work, for the first time a wireless treatment based on piezoelectric nanoparticles has been exploited to remotely deliver electric stimulations to breast cancer cells; chronic piezoelectric stimulation was able to upregulate the mature mRNA expression of *KCNJ6* gene encoding for Kir3.2 inward rectifier K^+^ channels, to affect Ca^2+^ homeostasis, and finally to induce the arrest of the cell cycle in G0/G1 phases.

In a previous work of our group, we estimated, thanks to a mathematical electroelastic model, as piezoelectric BTNPs undergoing US stimulation generate oscillating voltages in the order of magnitude of 1–10 mV, and we experimentally demonstrated as BTNPs can be successfully exploited as piezo-nanotransducers for the remote activation of neural cells^18^. In order to locally deliver low-intensity electric fields in proximity of HER-positive breast cancer cells (*e.g*., SK-BR-3), BTNPs have been functionalized with anti-HER2 antibody through streptavidin-biotin conjugation^33^.

Nanoparticle functionalization with anti-HER2 antibody is widely exploited in the literature, and was for example used in a work of the group of J.L. West to target near infrared (NIR)-absorbing gold nanoshells to HER-positive breast cancer cells^34^. In our work, we have observed a functionalization efficiency in terms of number of antibody molecules *per* BTNP of about 220, comparable to that obtained by the group of J.L. West (about 150 antibodies *per* gold nanoshell). Considering the surface of BTNPs and of nanoshells, the molecule number normalized to the surface area is, respectively, 785/μm^2^ for BTNPs and 3317/μm^2^ for gold nanoshells (about 4-fold difference). Specific geometric properties of the different nanomaterials, such as size^35^ and curvature^36^, are known to affect functionalization efficiency, therefore limiting the possibility of direct comparisons. However, despite the number of antibody molecules *per* surface unit is 4-fold lower for Ab-BTNPs with respect to Ab-functionalized gold nanoshells, both nanoparticles were able to successfully target and to strongly associate to SK-BR-3 cells.

Concerning our chronic piezoelectric stimulation, the anti-proliferative effects on SK-BR-3 cells were observed by using independent approaches and, in all cases, cell proliferation resulted to be not affected by the plain application of US. Although we cannot completely exclude that the US stimulation may alter the interaction between Ab-BTNP and HER2 receptors (*e.g*., with a local release of the antibody) with a consequent enhancement of the anti-proliferative effects, this scenario is rather unlikely because, as pointed out, the amount of antibody associated to the BTNPs is extremely lower than the typical therapeutic concentrations.

Supporting the hypothesis of piezoelectric stimulation, the mechanisms of proliferation inhibition triggered by the approach presented in our work display analogous features with respect to those induced by the direct application of electrical stimuli. Independent works demonstrate as low-intensity currents are able to reduce cell proliferation by upregulating the expression of K^+^ channels Kir3.2 and by affecting the cytoskeletal elements of the mitotic spindle^9–11^. Mitotic spindles are mostly characterized by microtubules consisting of protofilaments of tubulin heterodimers, polar structures with a static dipole moment. Contrary to the quiescence phase, during mitosis microtubules of mitotic spindle are spatially organized, display very large electric dipole moments^37^, and therefore their orientation can be affected by the presence of electric fields^11,38,39^.

Kir channels play an important role in cell proliferation, and their expression levels finely regulate the progression/arrest of cell cycle^40^. The inhibition of Kir channels in quiescent cells is known to increase their proliferation through G1/S checkpoint progression; moreover, a premature regulation of Kir induces the arrest in G1/G0 gap phases. Conversely, the arrest of cell cycle in S phase is associated to an increase of outward K^+^ currents and by a concomitant decrease of inward K^+^ currents^41^. For this reason, disruption of K^+^ fluxes can lead to the cell cycle arrest in different phases of the cycle. According to this mechanism, we observed both the upregulated expression of *KCNJ6* and the arrest of cell cycle in the G1/G0 phases (no significant mRNA level alteration of *KCNJ6* was instead detected in US-stimulated cultures without BTNPs). Coherently, Cucullo *et al*. exploited electrical stimulations on different cancer cell models like glioma (C6 line), prostate tumor (PC-3 cells) and lung tumor (H1299 cultures), and only cells that showed an upregulation of Kir3.2 channels (C6 and PC-3) underwent cell cycle arrest^10^. This stimulation has been tested by tuning different parameters (*i.e*., frequency and amplitude), and enhanced anti-proliferative effects were observed on Kir3.2 expressing cells by increasing both frequency and intensity until 50 Hz and 1.7 µA. Electrical stimulations were instead not able to affect proliferation levels and Kir3.2 channel expression in H1299 cells. Interestingly, ethanol exposure, a well-known risk factor for breast cancer, significantly decreases the expression of Kir channels in breast cancer cells^42^. Although a direct correlation between the two stimulation approaches (direct electric stimulation involves an electric field with alternated orientation that homogeneously stimulate the cell, while piezo-nanomaterial assisted-stimulation involves the local generation of an electric potential on the surface of nanoparticles undergoing US stimulation), an increased US power intensity is able to induce a proportional increase of voltage generated by piezo-nanoparticles^18^, and, consequently, a linearly enhanced activity of electrically-sensitive cells (as demonstrated in neurons)^24^. Coherently, in this work we have reported a trend of decreasing cell proliferation when stimulating BTNP-treated cultures with increasing intensity of US (from 0.2 to 1.0 w/cm^2^), despite only US intensity of 1 W/cm^2^ + BTNPs was successful in inducing a statistically significant inhibition of cell proliferation.

Similarly to electrical cues, also electromagnetic fields (EMF)-based stimulations have been exploited to limit cancer growth. In a recent work of Buckner *et al*., time-varying electromagnetic fields resulted able to reduce the proliferation of different malignant cell lines (including MDA-MB-231 breast adenocarcinoma cells) by increasing intracellular Ca^2+^ concentrations through voltage-gated T-type Ca^2^ channels, altering cyclin expression and delaying cell cycle progression^32^. Similarly to our work, 1 h/day of stimulation was enough to induce anti-proliferative effects. Chronic EMF stimuli were also successfully tested *in vivo* and resulted effective in reducing the growth of B16-BL6 cancer cells implanted in C57b mice.

## Conclusions

Nanoparticle-assisted chronic piezoelectric stimulation was adopted to inhibit cancer cell proliferation. The approach relies on targeting piezoelectric nanotransducers to cancer cells and thereafter to locally deliver an electric stimulation mediated by ultrasounds. The US-driven wireless cell stimulation, in principle, would allow inhibiting cancer cell proliferation without affecting the viability and the functions of healthy tissues. Future works will be focused, *in vitro*, to assess the anti-proliferative effectiveness of piezo-stimulation on other cancer cell types and, *in vivo*, to evaluate the accumulation/retention of the nanomaterial at the tumor site as well as the inhibitory effects of piezo-stimulation on cancer tissue growth. Moreover, despite the proposed nanosystem showed a successful targeting ability toward HER2-overexpressing breast cancer cells (the primary goal of this work), a detailed characterization of the antibody orientation, combined to the possibility to follow alternative functionalization strategies^43^, would allow further improving the nanomaterial targeting ability. Such modifications/characterizations, even if out of the scope of this work, will be particularly important for future *in vivo* investigations, where the efficiency of the nanomaterial delivery/targeting represents a fundamental parameter.

Finally, since drug resistance of cancer cells can be counteracted by applying chronic electric stimulations, piezoelectric nanomaterials will be included in multifunctional nanosystems able to target cancer cells, locally release anticancer drugs, and remotely deliver electrical stimulations, in order to both inhibit cancer growth and enhance the cytotoxic effects of chemotherapy drugs.

## Electronic supplementary material

Supplementary Information
