# Supplementary material for: Ultrasound-Activated Piezoelectric Nanoparticles Inhibit Proliferation of Breast Cancer Cells
Source: Sci Rep. 2018 Apr 19;8:6257. doi: 10.1038/s41598-018-24697-1 (PMC5908835; doi:10.1038/s41598-018-24697-1)
Supplement: Supplementary file 1 — Supplementary Information [file 41598_2018_24697_MOESM1_ESM.pdf]

Ultrasound-Activated Piezoelectric Nanoparticles Inhibit Proliferation of Breast Cancer Cells

SUPPORTING INFORMATION

Attilio Marino[1,*], *Matteo Battaglini*[1,2], *Daniele De Pasquale*[1,2], *Andrea Degl'Innocenti*[1], *Gianni Ciofani*[1,3,*]

[1]Istituto Italiano di Tecnologia, Smart Bio-Interfaces, Viale Rinaldo Piaggio 34, 56025 Pontedera, Italy

[2]Scuola Superiore Sant'Anna, The Biorobotics Institute, Viale Rinaldo Piaggio 34, 56025 Pontedera, Italy

[3]Politecnico di Torino, Department of Mechanical and Aerospace Engineering, Corso Duca degli Abruzzi 24, 10129 Torino, Italy

[*]attilio.marino@iit.it; gianni.ciofani@iit.it

Figure S1. (a) Hydrodynamic size (on the left) and *Z*-potential (on the right) of BTNP dispersion measured through dynamic light scattering. (b) TGA curves of DSPE-PEG (in black), plain BTNPs (powder, in red) and BTNPs coated with DSPE-PEG (in blue).

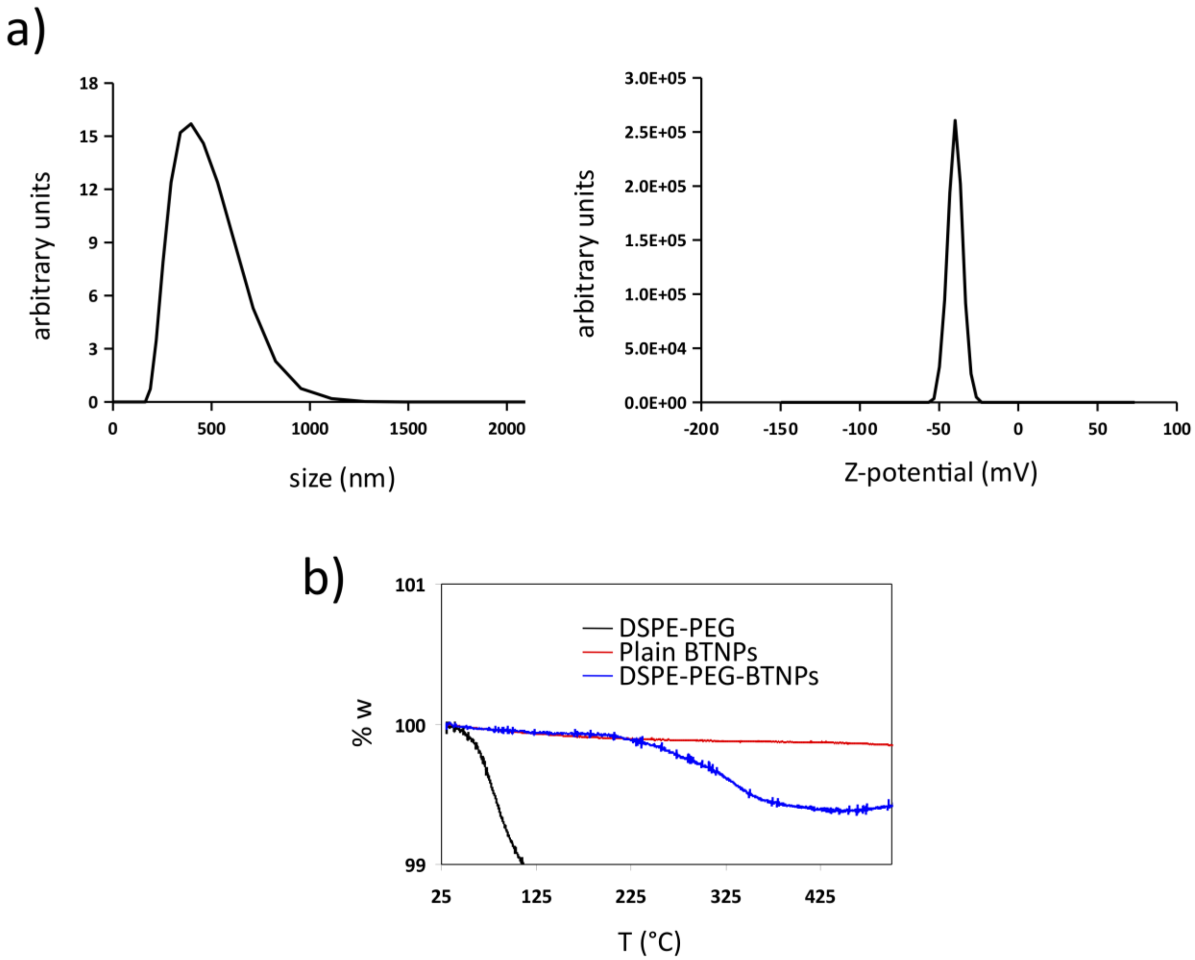

Figure S2. Metabolic activity (WST-1) of SK-BR-3 cells (a) treated for 3 days or 5 days with different concentrations of anti-HER2 (0.5-100 µg/ml), and (b) incubated for 5 days with BTNPs or Ab-BTNPs (10-250 µg/ml; * $p < 0.05$).

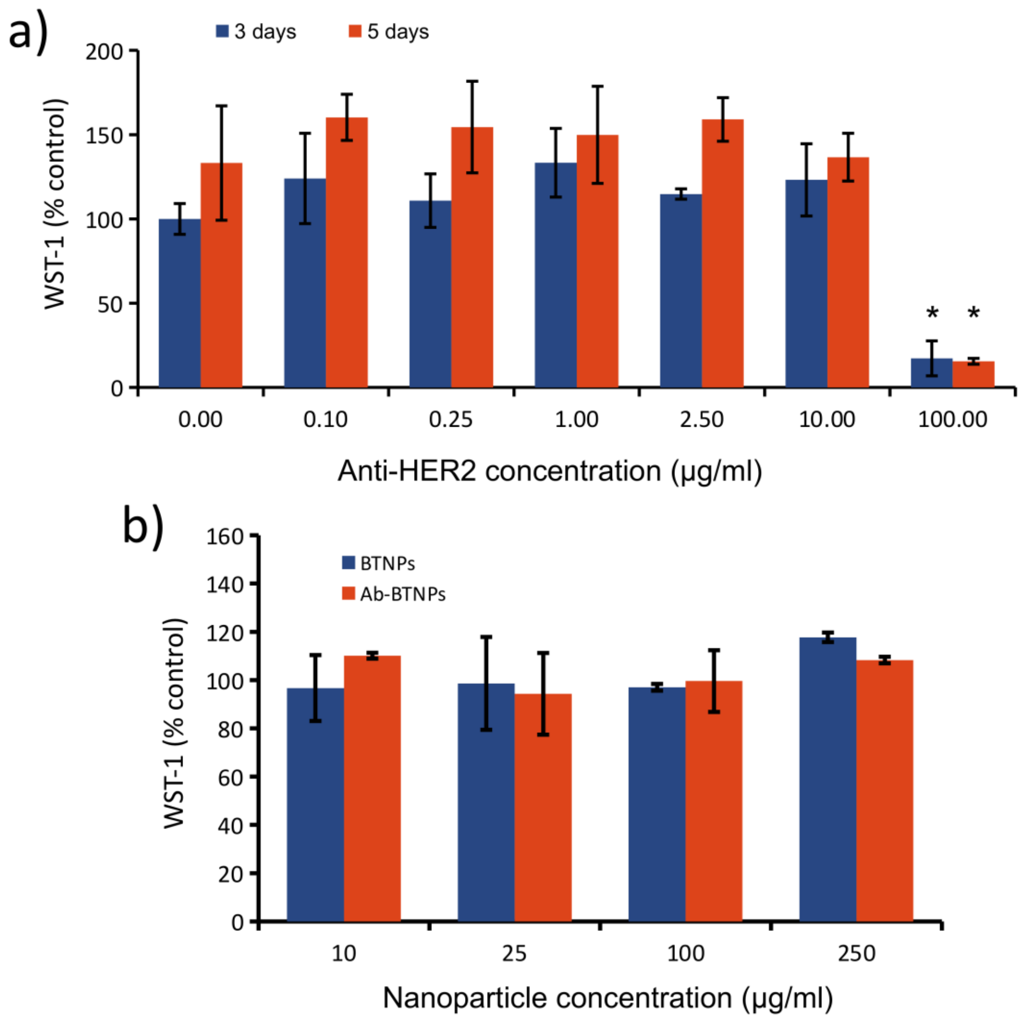

Figure S3. 3D rendering of confocal fluorescence images of SK-BR-3 cells treated with BTNPs or Ab-BTNPs (plasma membranes in green, nanoparticles in red).

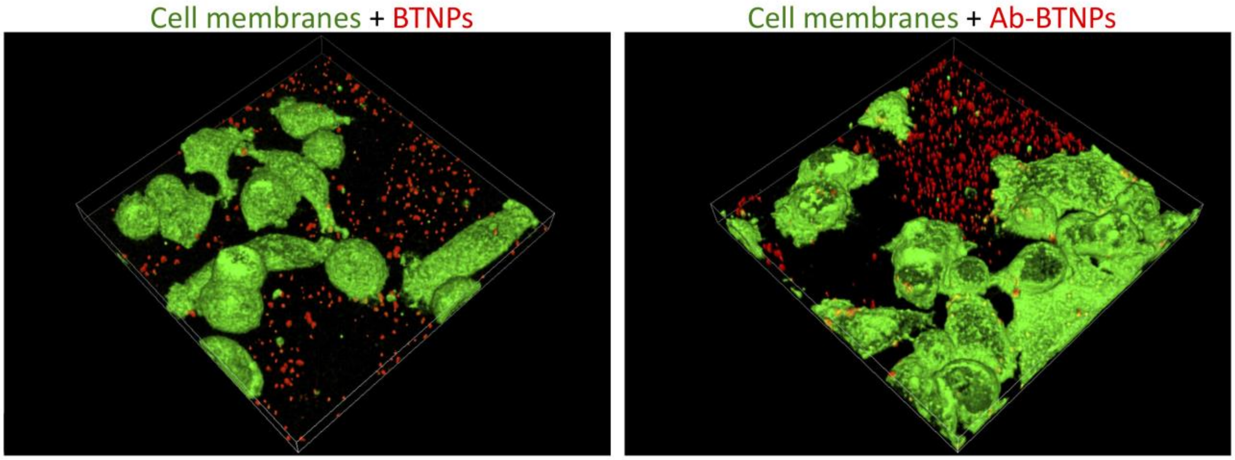

Figure S4. Scanning electron microscopy (SEM) images combined with energy-dispersive X-ray analysis (EDX) of BTNPs on SK-BR-3 cell membranes. (a) low-magnification and (b) high-magnification SEM of SK-BR-3 cells treated with BTNPs. (c) EDX performed on a portion of cell membrane associated to a cluster of nanoparticles (Ti and Ba are showed in pseudo-colors red and green, respectively). (d) Corresponding EDX spectrum confirming the presence of the characteristic peaks of Ba (4.47 and 4.83 keV) and of Ti (4.51 keV); other peaks are related to Au (sputtered on the sample), C and O.

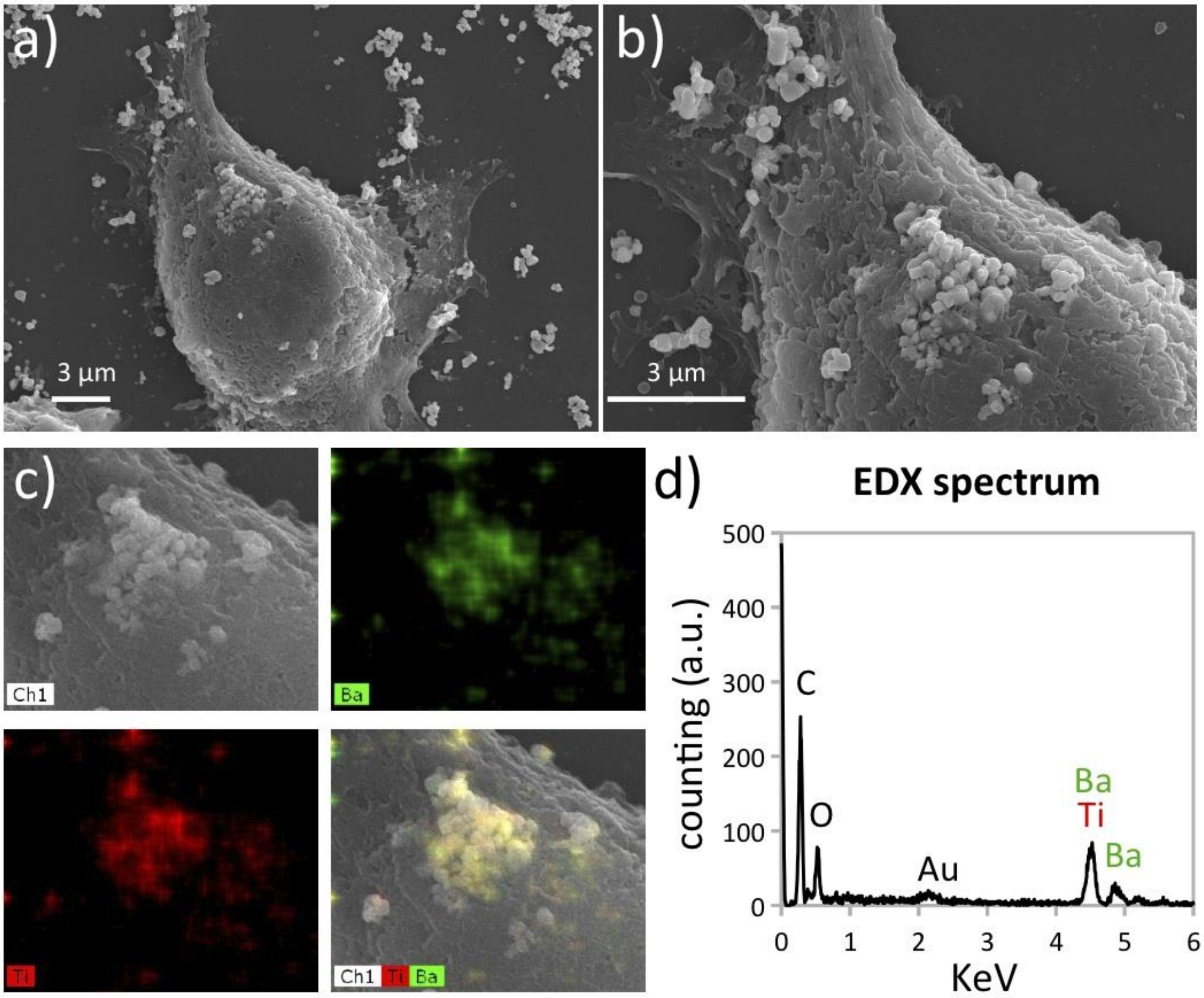

Figure S5. Confocal fluorescence images showing the internalization of BTNPs or Ab-BTNPs in SK-BR-3 cells at different time points (4 and 24 h); lysosomes in green, nanoparticles in red, nuclei in blue.

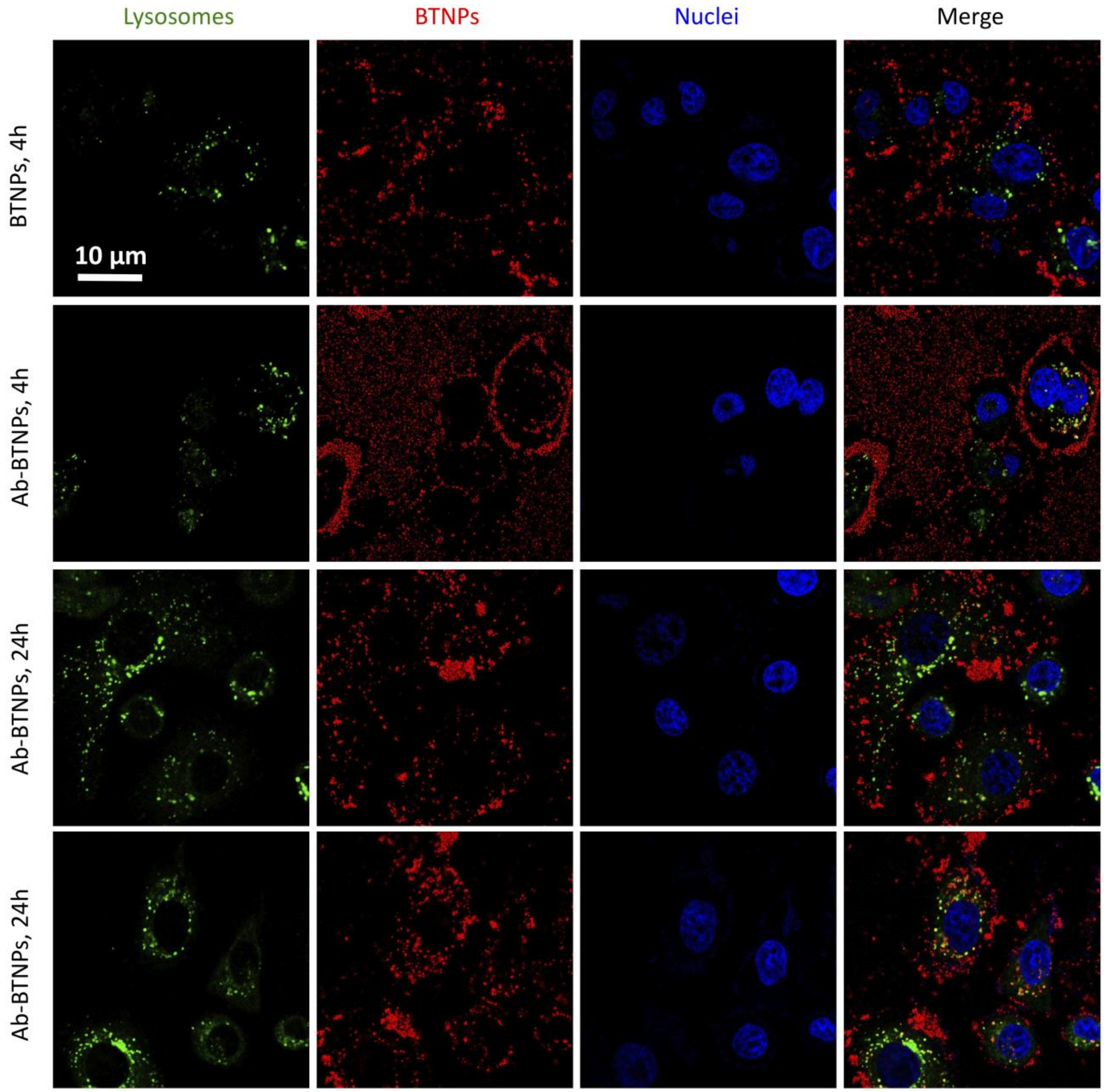

Figure S6. Metabolic activity (WST-1) of control cultures, ultrasound-stimulated cells without Ab-BTNPs (US) and cells stimulated with different US intensities (0.2 - 1.0 W/cm^2) in presence of Ab-BTNPs (US+BTNPs). *$p < 0.05$.

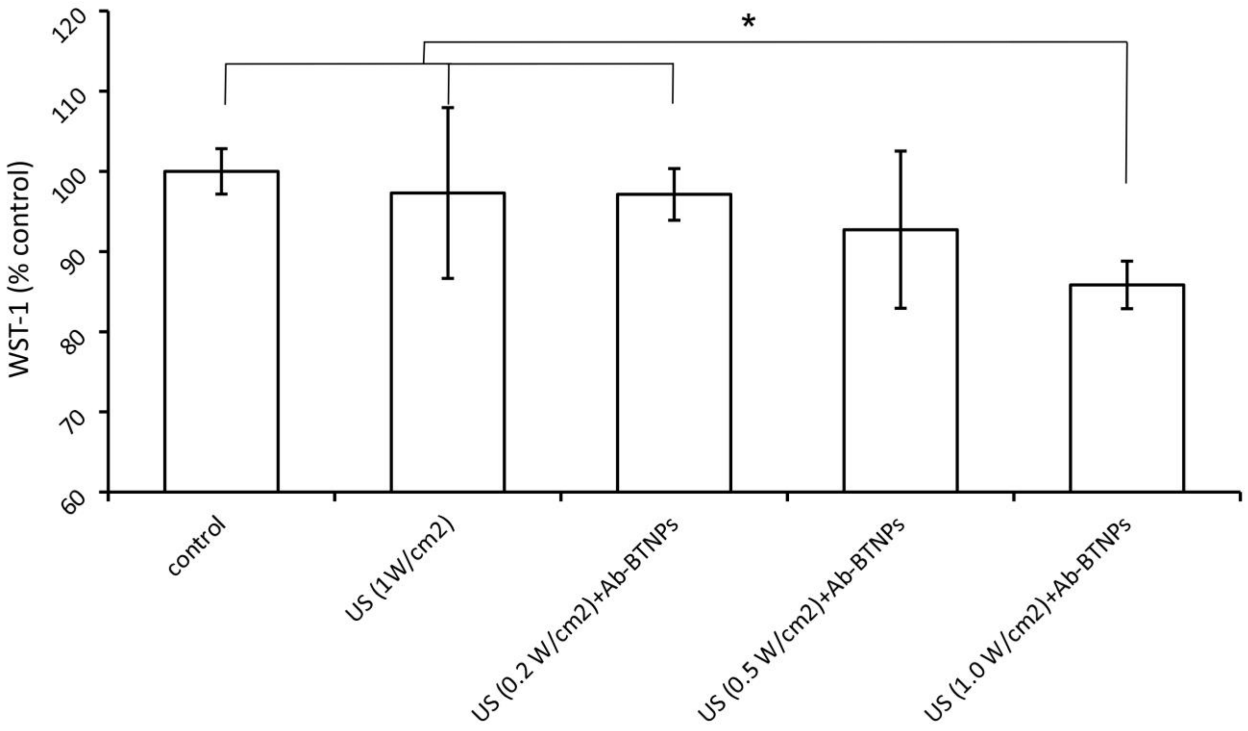

Table S1. qPCR primers for *KCNJ6* and *RPL32*. Primer sequences are reported 5’-3’.

Gene	Primer F	Primer R
KCNJ6	TTGCATTTCCTTCCCTCGCC	TTGTGCTTTTCCTGGCTGCG
RPL32	GAAGTTCTTGGTCCACAACG	GAGCGATCTCGGCACAGTA
